# The emerging pathogen *Candida auris*: host interactions and disease drivers

**DOI:** 10.1186/s12929-026-01230-5

**Published:** 2026-02-21

**Authors:** Daniel Ruben Akiola Sanya, Ashley Valle Arevalo, Djamila Onésime, Clarissa J. Nobile

**Affiliations:** 1 INRAE, AgroParisTech, Micalis Institute, Université Paris-Saclay, 78350 Jouy-en-Josas, France; 2 Department of Molecular and Cell Biology, University of California, Merced, Merced, USA; 3 Health Sciences Research Institute, University of California, Merced, Merced, USA

**Keywords:** Diagnostic markers, Fungal infection, Clinical microbiology, Antifungal agents, *Candida auris*, Antimicrobial resistance, Fungi, Pathogens, Cellular microbiology

## Abstract

*Candida auris* has emerged over the past fifteen years as a multidrug-resistant human fungal pathogen responsible for hospital-acquired infections associated with high mortality rates. Clinical isolates frequently exhibit resistance to one or more first-line antifungal drugs, and management is further complicated by persistent colonization, biofilm formation, and historical misidentification in diagnostic laboratories. Rapid species-level identification and accurate antifungal susceptibility testing are critical for effective patient care and infection control. In this article, we summarize the clinical spectrum of *C. auris* infections, highlight key pathogenic traits and clade-specific biological differences, and discuss emerging diagnostic, therapeutic, and preventative strategies—including novel antifungal agents, host-directed therapies, and vaccine development—that may improve detection and expand treatment options for *C. auris* infections.

## Introduction

*Candida auris* has emerged over the past decade as a multidrug-resistant human fungal pathogen responsible for hospital-acquired infections associated with high mortality worldwide. Its rapid global emergence, combined with resistance to multiple antifungal drug classes, poses a serious and growing threat to public health [1, 2]. Since its first identification, *C. auris* outbreaks have been reported in healthcare facilities on five continents and in at least 50 countries [1, 3], including the United States [3], the United Kingdom [4], Spain [5], Venezuela [6], Singapore [7], Italy [8], Colombia [9], Kuwait [10], and Kenya [11]. Clinical manifestations range from superficial mucosal infections to invasive bloodstream infections, which are associated with mortality rates of approximately 30–60% [12]. Notably, *C. auris* can co-infect with *Candida albicans* in conditions such as vulvovaginal candidiasis (VVC), where these polymicrobial interactions may exacerbate disease severity relative to *C. albicans* infection alone [13].

Several biological features distinguish *C. auris* from other *Candida* species and contribute to its persistence in healthcare environments. *Candida auris* can survive on hospital surfaces for extended periods (often weeks) [14], tolerates hypersaline conditions (salt concentrations higher than seawater) [15], and withstands elevated temperatures up to 42 °C [1, 16]. These traits facilitate efficient colonization of human skin and environmental reservoirs, rendering eradication particularly challenging once *C. auris* is established. Mechanistically, the Hog1 mitogen-activated protein kinase pathway has been identified as a critical regulator of skin colonization and intradermal persistence [17]. In parallel, the adhesin Als4112 has emerged as a major determinant of host interaction across all major clades (I–IV). Als4112 is both necessary and sufficient for strong keratinocyte adherence, promotes binding to extracellular matrix components such as skin laminin, and enhances fungal burden on the skin, collectively contributing to systemic virulence [18].

Transmission of *C. auris* most commonly occurs in healthcare settings through contact with contaminated surfaces or equipment [1], and potentially through ventilation systems [19]. A recent report described a probable case of patient-to-patient transmission in a burn intensive care unit following a prolonged incubation period of approximately two months [20]. The ability of *C. auris* to persist within patients and hospital environments raises particular concern, as prolonged residence may facilitate adaptive responses to antifungal pressure. Supporting this idea, one study found that “older” *C. auris* cells (cultured for ten generations) exhibited greater tolerance to antifungal drugs including fluconazole, micafungin, 5-flucytosine, and amphotericin B compared to “younger” *C. auris* cells (cultured for three or fewer generations) [21]. In addition, *C. auris* has recently been detected on fungicide-treated apples exposed to agricultural demethylation inhibitors targeting sterol biosynthesis, raising the possibility that environmental fungicide use may contribute to the emergence or selection of azole-resistant strains [22].

From a diagnostic perspective, *C. auris* presents additional challenges due to its close phenotypic and phylogenetic relatedness to the *Candida haemulonii* species complex, which includes *C. haemulonii*, *Candida duobushaemulonii*, *Candida pseudohaemulonii*, *Candida haemulonii* var. *vulnera*, and *Candida vulturna*. As a result, *C. auris* is frequently misidentified using conventional clinical laboratory methods and has also been mistaken for other yeasts such as *Rhodotorula glutinis, Candida sake*, *Candida lusitaniae*, and *Candida parapsilosis* [23, 24]. These diagnostic limitations are especially problematic given the rising incidence of *C. auris* infections among critically ill patients, particularly those requiring ventilator support or tracheostomies, who are at increased risk of colonization [25]. Early and accurate identification is therefore essential for effective patient management, outbreak surveillance, and infection control.

To date, six genetically distinct clades of *C. auris* have been described [26–28], designated as clade I (South Asia), clade II (East Asia), clade III (Africa), clade IV (South America), clade V (Iran), and clade VI (Indomalaya/Singapore) [27–29]. Distinguishing between these clades remains technically challenging. Current diagnostic strategies typically rely on culture-based methods followed by biochemical assays and/or DNA sequencing targeting the internal transcribed spacer (ITS) region and the 28S rRNA gene [30]. While useful, these approaches often suffer from limited sensitivity, prolonged turnaround times, high costs, or the need for specialized equipment, and can fail to reliably differentiate *C. auris* from closely related species such as *C. haemulonii* [31]. Combining ITS and 28S rRNA sequencing with biochemical platforms such as the VITEK 2 YST system can improve identification accuracy [32]. In addition, matrix-assisted laser desorption/ionization time-of-flight mass spectrometry (MALDI-TOF MS) has emerged as a rapid and cost-effective method for identifying *C. auris* from pure cultures, provided that reference databases are properly curated and updated [33–35]. Nonetheless, even with current MALDI-TOF MS databases (e.g., VITEK version 8.01), challenges remain in distinguishing *C. auris* from *C. duobushaemulonii* and in resolving certain clades, particularly clades I–III [7, 36, 37].

In the following sections, we review the spectrum of infections caused by *C. auris*, examine key pathogenic traits that shape its interactions with the host, and discuss clade-specific biological differences among clinical isolates. We further highlight emerging diagnostic, therapeutic, and preventive strategies that may improve their detection, management, and long-term control.

## Infections caused by *C. auris*

Emerging fungal pathogens represent an increasing threat to human health, driven by rising antifungal resistance and high associated mortality rates[1, 38, 39]. Among these pathogens, *C. auris* has garnered particular concern due to its multidrug resistance, elevated mortality, exceptional environmental persistence—surviving on surfaces for weeks to months—and its capacity to tolerate hypersaline conditions and elevated temperatures [1, 16, 38].

*Candida auris* infections occur predominantly in individuals with established risk factors for invasive fungal disease. These include patients undergoing hemodialysis, receiving prolonged antifungal therapy, or requiring mechanical ventilation, as well as those with implanted medical devices such as indwelling central venous catheters [4, 9, 11, 40]. Underlying conditions including lymphoma, HIV infection, chronic kidney disease, hypertension, and diabetes further increase susceptibility. Epidemiological studies indicate a higher incidence in men compared to women, with premature neonates and elderly individuals representing particularly vulnerable populations [41]. Notably, *C. auris* coinfections have been increasingly reported in patients with severe acute respiratory syndrome coronavirus 2 (SARS-CoV-2) infection during the COVID-19 pandemic, underscoring its opportunistic nature in critically ill hosts [42–46].

A defining feature of *C. auris* pathogenicity is its ability to form biofilms, a well-established driver of persistent and difficult-to-treat *Candida* infections [47]. Similar to other *Candida* species, *C. auris* readily adheres to abiotic and biotic surfaces and develops biofilms that exhibit pronounced resistance to azoles and amphotericin B [48, 49]*.* Biofilm-associated cells upregulate genes encoding drug efflux pumps, further enhancing antifungal tolerance and resistance [50, 51]. Clinically, biofilms promote long-term environmental survival and facilitate persistence within the host, where they can serve as reservoirs for invasive disease. Consistent with this, a retrospective study of intensive care unit (ICU) patients in Pakistan identified a strong association between indwelling catheters and the development of *C. auris* bloodstream infections [52].

Biofilm development in *C. auris* progresses through distinct stages, beginning with surface adherence by planktonic yeast cells, followed by proliferation, maturation, and eventual dispersal [53]. Unlike *C. albicans*, which undergoes extensive filamentation, mature *C. auris* biofilms are composed predominantly of yeast-form cells arranged in dense, structured clusters. During advanced stages of biofilm development, *C. auris* releases dispersed yeast cells into the surrounding environment, facilitating colonization of new niches and perpetuating the biofilm lifecycle. In *C. albicans*, biofilm-dispersed cells originating from vascular catheters can directly enter the bloodstream, leading to severe candidemia [54]. These dispersed cells exhibit enhanced adhesion to and damage of endothelial cells, increased virulence, heightened antifungal resistance, and an augmented capacity to form secondary biofilms compared to planktonic cells [55]. Recent studies indicate that *C. auris* biofilm-dispersed cells share many of these pathogenic traits. *Candida auris* cells overexpress genes associated with biofilm initiation and maturation, including *ALS5* and *KRE6*, and display a distinct metabolic profile that enhances survival under stress and nutrient-limited conditions [56]. Notably, these cells show increased expression of antifungal resistance-associated genes (*ERG2, ERG6, ERG11, FKS1, CHS1, CHS2, CDR1,* and *MDR1*) relative to their planktonic counterparts. Metabolically, dispersed cells are enriched in glyoxylate and dicarboxylate pathways, as well as glycerolipid metabolism, reflecting a high degree of metabolic plasticity, whereas planktonic cells are preferentially enriched in fatty acid biosynthesis pathways [56].

Collectively, these clinical and mechanistic observations underscore how *C. auris* not only causes severe and treatment-refractory infections but also establishes persistent interactions with both the host and the environment—features that underpin its success as an emerging nosocomial pathogen and are explored further in the following section.

## *Candida auris* features associated with host pathogenesis

### Host immune recognition and immune evasion strategies of *C. auris*

Host innate immune recognition of *C. auris* is complex and remains incompletely understood. Conflicting reports describe both heightened and attenuated proinflammatory responses relative to *C. albicans* [24, 57–61]. These discrepancies likely reflect differences in host immune status, infecting clade or strain, fungal morphotype, and experimental models. Despite evidence of innate immune activation, *C. auris* exhibits a remarkable capacity to persist within host immune cells and evade clearance.

Compared to *C. albicans*, *C. auris* can induce elevated levels of proinflammatory cytokines, including TNF-α, IL-6, and IL-1β, indicative of innate immune activation [57]. Paradoxically, however, *C. auris* demonstrates reduced macrophage lysis and attenuated virulence in murine models of disseminated infection [57]. One explanation lies in its distinctive cell wall architecture: *C. auris* mannans mask underlying β-glucans, limiting recognition by the pattern-recognition receptor Dectin-1 [59]. These mannans differ structurally from those of *C. albicans*, featuring lower molecular mass acid-labile α-1,2-mannose-phosphate side chains that reduce engagement of additional C-type lectin receptors (CLRs), such as Dectin-2 and mannose-binding lectins [57]. Although *C. auris* engages host CLRs including CR3 and the macrophage mannose receptor (MMR) to stimulate cytokine production, these responses are generally less robust than those elicited by *C. albicans*.

Beyond altered immune recognition, *C. auris* actively evades immune-mediated killing. It can replicate within macrophages without inducing significant host cell lysis [62] and resists neutrophil-mediated killing [53, 66]. In addition, *C. auris* releases extracellular vesicles (EVs) that activate the STING pathway [63] while simultaneously enhancing fungal adhesion to epithelial cells [64], suggesting a dual role in immune modulation and tissue colonization. Exposure to *C. auris* also activates type I and type II interferon signaling pathways, including interferon-stimulated genes such as *ISG15* [57], further shaping host immune responses.

Consistent with its ability to persist within host immune cells, transcriptomic analyses reveal extensive metabolic reprogramming during host–pathogen interactions. *Candida auris* upregulates genes involved in alternative carbon metabolism, nutrient transport, proteolysis, oxidative stress resistance, and cell wall biosynthesis [62, 65]. Ex vivo whole-blood infection models further show increased expression of genes associated with chaperone-mediated protein folding (e.g., *HSP6*), ergosterol biosynthesis (e.g., *ERG2*, *ERG6*), chitin synthesis (e.g., *CHS3*, *CHS8*), transmembrane transport (e.g., *PHO84*, *HGT10*, *OPT2*), and antioxidant defenses (e.g., *PRX1*) [65]. In contrast, genes involved in fatty acid β-oxidation (e.g., *CRC1*, *POX1–3*) are downregulated, indicating a metabolic shift away from fatty acid catabolism during host infection [65, 66].

A striking manifestation of this metabolic adaptation is the formation of giant lipid droplet (gLD)-containing cells, which represent a specialized survival state in *C. auris* [67]. gLD-containing cells exhibit markedly thickened cell walls, sustained mitochondrial activity, and upregulation of pathways involved in acyl-CoA and triacylglycerol synthesis (e.g., *ACS1*, *ACS2*, *FAA2–3*, *PDA1*). These cells also show enhanced expression of genes involved in iron acquisition (e.g., *SIT1*, *FRE9*, *FRP1*), ergosterol and sphingolipid biosynthesis (e.g., *ERG24*, *ERG6*, *ERG3*, *SUT1*, *MTS1*), cell wall remodeling (e.g., *KRE9*, *ENG1*, *XOG1*, *CHS2*, *CHS3*), and glucose transport (e.g., *HGT19*, *HGT7*, *HGT2*, *HGT13*). Conversely, pathways involved in amino acid biosynthesis, transport, and protein translation are downregulated.

Biochemically, gLD-containing cells accumulate elevated levels of fatty acids, triacylglycerols, sterols, phosphatidylserines, and glycosphingolipids, while exhibiting reduced levels of polyunsaturated phospholipids. Functionally, these cells display enhanced skin colonization capacity, increased resistance to environmental stressors and antifungal drugs, including amphotericin B, and heightened tolerance to host-derived antimicrobial peptides such as LL-37 and PACAP [68–71]. Together, these features indicate that gLD formation represents an adaptive strategy that promotes persistence on human skin and survival in hostile host environments.

Consistent with these observations, *C. auris* undergoes extensive sphingolipid remodeling, with sphingolipid profiles varying across clades and antifungal resistance phenotypes [72]. High-resolution lipidomics has identified over 140 sphingolipid species spanning nine classes, with phytoceramides dominating the sphingolipid landscape. Drug-resistant isolates—particularly fluconazole-resistant strains—exhibit elevated SL abundance and greater fatty acyl diversity compared to amphotericin B-resistant isolates, generating resistance-specific lipid “fingerprints.” Notably, despite this diversity, *C. auris* remains broadly susceptible to inhibitors of sphingolipid biosynthesis, such as myriocin and aureobasin A, highlighting sphingolipid metabolism as a promising therapeutic target [72].

**Fig. 1:**
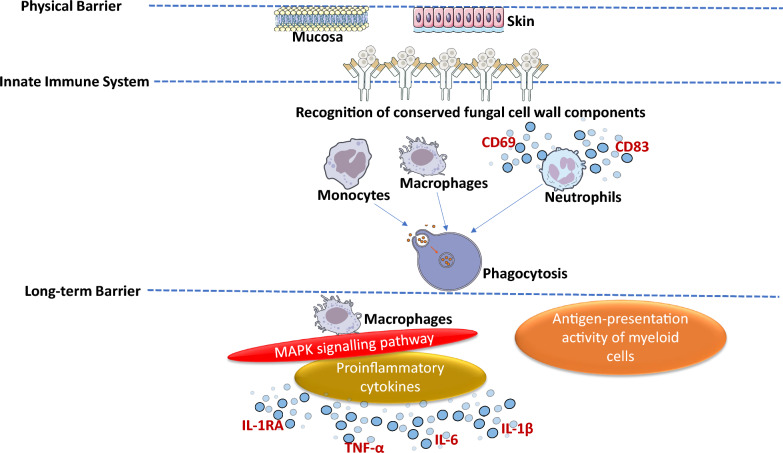
Overview of host immune system barriers implicated in protecting the host against *C. auris* infection. Three major types of host immune system barriers (physical, innate immune, and long-term) are involved in the host response to *C. auris*. This figure summarizes information from [57, 59, 65, 195]

Collectively, these immune evasion, metabolic, and lipid remodeling strategies underscore the remarkable adaptability of *C. auris* within the host. By integrating altered immune recognition with intracellular survival and profound metabolic flexibility, *C. auris* establishes persistent colonization and resists immune-mediated clearance, contributing to its success as an emerging multidrug-resistant pathogen. An overview of host immune barriers and fungal evasion strategies is summarized in Fig. 1.

### Antifungal resistance and host pathogenesis

Antifungal resistance is a defining feature of *C. auris* pathogenicity and a major contributor to its clinical impact. Most isolates exhibit secondary resistance to fluconazole, with variable resistance to amphotericin B and echinocandins reported across clades [73, 74]. Recent work has revealed that dermal-tropic *C. auris* isolates can acquire amphotericin B resistance through modulation of the carbon dioxide sensing pathway, mediated by the carbonic anhydrase Nce103 and the transcription factors Rca1 and Efg1. This resistance mechanism relies on the enzymatic conversion of carbon dioxide to bicarbonate, sustaining mitochondrial bioenergetics that are essential for persistence, skin colonization, and virulence in nutrient-limited host niches. Notably, within cutaneous microenvironments, hospital-associated bacterial pathogens such as *Proteus mirabilis* and *Klebsiella pneumoniae* produce carbon dioxide via urease activity, indirectly promoting *C. auris* fitness and dermal colonization [75]*.* These findings highlight how resistance mechanisms intersect with host- and microbiota-derived factors to support *C. auris* persistence.

Given the limited antifungal drug arsenal, dissecting the molecular basis of *C. auris* resistance is essential for improving treatment outcomes. One key contributor to resistance is biofilm formation, which creates a protective niche that limits antifungal drug penetration, impairs immune clearance, and enhances survival under environmental stress [76]. Biofilms serve as reservoirs for chronic infections and can seed bloodstream infections. Yet, how host immune responses differ across *C. auris* strains—and how these responses contribute to morbidity and mortality—remains incompletely understood [30, 77, 78].

At the genetic level, multiple resistance mechanisms have been characterized. Azole resistance commonly arises from mutations in *ERG11* (e.g., Y132F and K143R) [79] and in *TAC1B*, which encodes a zinc-cluster transcription factor regulating drug efflux [80]. Echinocandin resistance is associated with mutations in *FKS1* hotspot 1 (S639F, S639Y, and S639P) [81–83], which have been linked to recent cases of therapeutic failure and can be selected in vivo, including during urinary tract infections [83]. Rapid molecular diagnostics, such as TaqMan-based fluorescence melt curve PCR, enable detection of clinically relevant *FKS1* mutations (e.g., F635C/Y/del, S639F/Y/P), facilitating early resistance surveillance [84].

Advances in antifungal susceptibility testing (AFST) have further clarified resistance detection in *C. auris*. Reference broth microdilution (BMD) methods following the Clinical and Laboratory Standard Institute (CLSI) and the European Committee on Antimicrobial Susceptibility Testing (EUCAST) guidelines remain the gold standard, although standardized clinical breakpoints are still lacking [85]. Commercial platforms—including VITEK 2, Sensititre YeastOne, Etest, and MICRONAUT-AM—show method-dependent variability, particularly for amphotericin B and fluconazole. VITEK 2 frequently overestimates amphotericin B resistance while underestimating fluconazole resistance in some clades [86], whereas Sensititre YeastOne and Etest similarly tend to overestimate amphotericin B minimum inhibitory concentrations (MICs) [85, 87]. In contrast, echinocandin susceptibility is generally consistent across platforms, with low resistance rates reported (e.g., 0–3.67% for anidulafungin) [88]. These discrepancies underscore the need for cautious interpretation of commercial AFST results, use of method-specific epidemiological cutoff values, and confirmatory reference testing to guide therapy and surveillance [2].

New resistance mechanisms continue to emerge. For example, novel mutations in *ERG4* (M192I) and *ERG5* (A870C), conferring resistance to multiple antifungal classes, were identified during a *C. auris* outbreak among COVID-19 patients in Qatar [89]. Alterations in the ergosterol pathway also drive amphotericin B resistance, with mutations in *ERG3*, *ERG6*, *ERG10*, *HMG1*, and *NCP1* leading to sterol intermediates that reduce drug binding [90]. In some cases, chromosomal aneuploidies (e.g. in chromosomes 4 and 6) have been linked to amphotericin B resistance [90].

Computational approaches are further expanding the resistance landscape. Machine learning has predicted novel resistance-associated mutations in *C. auris*, such as Y501H and I466M in *ERG11*, and R278H in *ERG10*, that may be associated with fluconazole, micafungin, and amphotericin B resistance, respectively [91]. Similarly, genome-wide association studies (GWAS) have linked mutations in RNA-dependent DNA polymerases, zinc-binding transcription factors, and mitochondrial ribosomal proteins to azole and amphotericin B resistance [92]. These findings highlight new candidates for experimental validation.

Beyond single-gene mutations, resistant *C. auris* isolates exhibit broader physiological adaptations. Echinocandin-resistant strains display increased mannan and β-glucan levels, altered cell wall adhesins, and transcriptional rewiring of mitochondrial pathways [93]. Mutations in *FKS1* hotspot 2, such as R1354H, are particularly significant, as reverting these mutations to the wildtype, restores caspofungin susceptibility [94]. Resistance can also arise via efflux pump upregulation or aneuploidy-driven gene dosage effects [95].

**Fig. 2:**
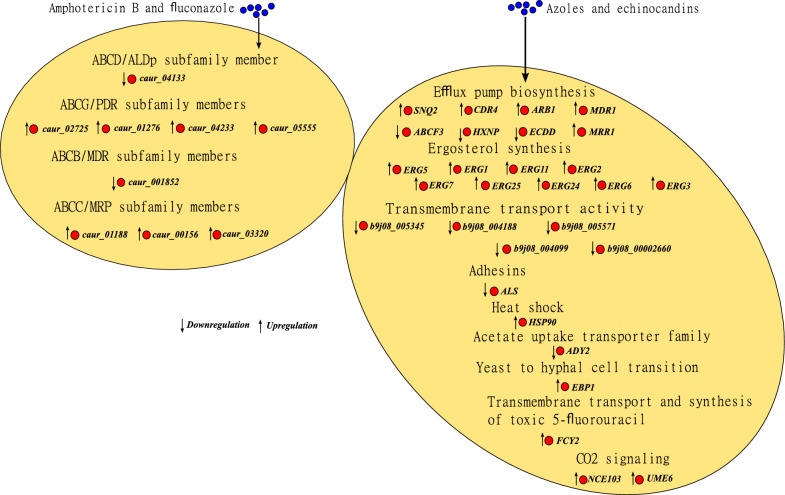
Overall regulation of genes involved in the antifungal drug response in multidrug-resistant strains of *C. auris*. (A) Multidrug resistant compared to susceptible strains subjected to the combination of amphotericin B and fluconazole. (B) Multidrug resistant compared to susceptible strains subjected to azoles and echinocandins. This figure summarizes information from [97] and [98]

**Fig. 3:**
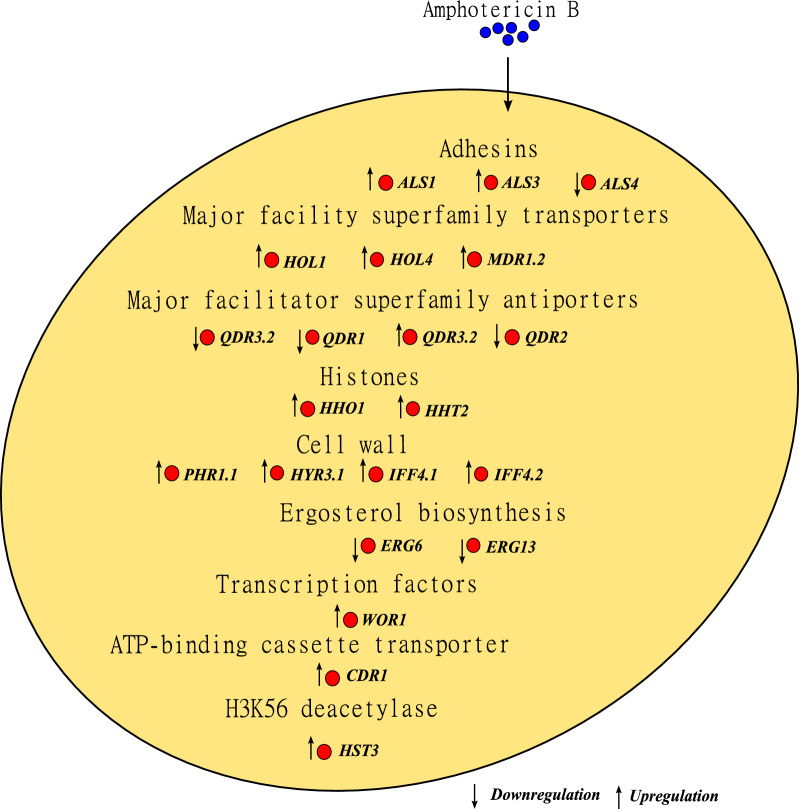
Overall regulation of genes involved in the antifungal drug response of amphotericin B-resistant strains of *C. auris*. Amphotericin resistant compared to susceptible strains of *C. auris* display the depicted gene expression patterns. This figure summarizes information from [196]

Omics-based studies provide further insight into antifungal resistance mechanisms. Proteomic analyses of azole-resistant *C. auris* strains reveal upregulation of mitochondrial proteins involved in respiration (e.g., Cox2, Cox12) and carbon metabolism (e.g., Gpm1, Ald5, Adh2) [96]. Transcriptomic comparisons of resistant versus susceptible isolates highlight differential expression of genes related to ABC transporters, drug efflux pumps, ergosterol biosynthesis, and stress response pathways [97, 98]. Notably, twenty putative *C. auris* ABC transporters show altered expression upon exposure to antifungal drugs [98]. These findings underscore the multifactorial nature of *C. auris* resistance, spanning mutations, transcriptional reprogramming, and metabolic shifts. To provide an overview of known resistant transcriptomic profiles, Fig. 2 summarizes gene expression patterns in multidrug-resistant versus susceptible *C. auris* strains exposed to two or more major antifungal drugs, while Fig. 3 focuses specifically on amphotericin B-resistance profiles.

Genetic engineering tools have accelerated mechanistic discovery. *Agrobacterium*-mediated transformation and CRISPR-Cas9 systems have enabled targeted mutagenesis, uncovering key roles for *ACE2*, *TAO3*, and *ELM1* in morphogenesis, virulence, and antifungal susceptibility [99]. More recently, CRISPR-Cas9 ribonucleoprotein systems revealed that Erg3 promotes synthesis of toxic sterols, with *ERG3* loss conferring azole resistance [100].

Finally, novel regulatory elements are emerging as resistance modulators in *C. auris*. A long non-coding RNA (lncRNA), *DINOR*, named for DNA damage-inducible non-coding RNA, was identified as a virulence factor and stress response regulator in *C. auris* [101]. Deletion of *DINOR* caused DNA damage and the upregulation of DNA repair systems, DNA replication, and morphogenesis, suggesting that *DINOR* plays a role in maintaining genome integrity. In addition, *C. auris* cells treated with amphotericin B or caspofungin were found to upregulate *DINOR*. Overall, little is known about the roles of lncRNAs in mediating antifungal resistance and virulence in the *Candida* species, and this could be an exciting area for future therapeutic development.

### Morphogenesis and phenotypic switching

Like other *Candida* species, *C. auris* undergoes distinct morphological transitions that contribute to its pathogenicity. Compared to *C. albicans*, *C. auris* forms fewer true hyphae and more pseudohyphae under standard filamentation-inducing conditions [102]. Many *C. auris* clinical isolates form aggregates of pseudohyphal cells that tolerate higher antifungal drug concentrations than non-aggregating isolates [1]. Intriguingly, these aggregating cells display reduced virulence in the *Galleria mellonella* infection model [60, 102–104], yet exhibit enhanced survival and increased brain colonization in murine models of disseminated candidiasis [105]. This apparent paradox highlights the niche- and host-dependent nature of *C. auris* virulence, suggesting that aggregation may favor persistence rather than acute pathogenicity.

Two primary mechanisms underlie aggregation in *C. auris*: (1) defects in cell division or daughter cell separation, and (2) increased cell–cell and cell-surface adhesion and enhanced biofilm formation [106–108]. The latter phenotype is mediated, in part, by variable copy numbers of the subtelomeric adhesin-encoding gene, *ALS4*. Differences in *ALS4* copy number and expression influence biofilm formation, surface colonization, and virulence, underscoring the contribution of adhesin variability to phenotypic diversity in *C. auris* populations [106].

Recent studies further demonstrate that *C. auris* virulence traits—including antifungal tolerance, stress adaptation, and growth fitness—are shaped by dynamic and reversible cellular states. These states enable frequent switching between “white” and “brown” morphotypes in response to environmental cues such as temperature and carbon source availability. This phenotypic plasticity is governed by transcriptional regulators including Wor1, Msn4, Crz2, Rca1, and Efg1 [109], reinforcing the idea that *C. auris* adapts rapidly to fluctuating host and environmental conditions.

### Enzymatic mediators of virulence

Several enzymatic virulence mechanisms first described in *C. albicans* also contribute to *C. auris* pathogenicity*.* Among these are extracellular hydrolytic enzymes such as secreted aspartyl proteinases (Saps), which promote host cell invasion [110]. In *C. auris*, Sap activity is regulated by the Ras/cAMP/PKA signalling pathway through its catalytic subunits Tpk1 and Tpk2 [111]. Comparative analyses identified fourteen proteins containing aspartic peptidase domains in *C. auris*, and functional studies of seven Saps (Saps1–7) revealed Sap3 as a major contributor to virulence and biofilm formation [111]*.*

In addition to Saps, *C. auris* produces copper-only superoxide dismutases (Cu-only Sods), a class of extracellular enzymes that detoxify reactive oxygen species and protect the fungus from the oxidative burst of host immune cells [112, 113]. A recent study also identified the Tsa1b peroxiredoxin, which is upregulated under oxidative stress and plays a dual role in oxidative stress resistance and host infection [114]. In *C. albicans*, the Cu-only Sods Sod4 and Sod5 are catalytically active—Sod5 being the most extensively studied—while Sod6 remains less characterized [115, 116]. *Candida auris* encodes two Cu-only Sod-like proteins: one homologous to *C. albicans* Sod4/5 and another homologous to Sod6 [116]. Notably, the *C. auris* Sod4/5-like enzyme is enzymatically active under iron-limited conditions, highlighting its potential role in adaptation to host nutrient restriction and positioning it as a promising target for antifungal drug development.

Together, morphological plasticity, phenotypic switching, and enzymatic defenses form an integrated virulence strategy in *C. auris*. By coupling flexible cellular states with robust oxidative stress resistance and proteolytic capacity, *C. auris* enhances its ability to persist within host tissues, evade immune clearance, and establish chronic or disseminated infection.

## Clade-specific differences among *C. auris* clinical isolates

*Candida auris* isolates are divided into four main clades (clades I, II, III, IV), a rarer clade V [117, 118], and a more recently reported clade VI [119, 120]. These clades correspond to distinct evolutionary lineages and geographic origins and differ markedly in genetic composition, metabolic capacity, virulence traits, and antifungal susceptibility. As such, clade tracking has become an increasingly valuable tool in outbreak investigations, enabling discrimination between local transmission events and new introductions, and informing targeted public health responses.

This utility of clade-resolved genomic surveillance was demonstrated during a prolonged *C. auris* outbreak in Nevada, where whole-genome sequencing of approximately 200 clinical isolates revealed genetically distinct subgroups within clades I and III based on shared single-nucleotide polymorphism (SNP) ancestry [121]. This approach enabled investigators to distinguish ongoing intra-facility transmission from independent introductions and facilitated the early identification of isolates originating outside the state [121].

Beyond epidemiology, substantial metabolic and phenotypic heterogeneity exists among *C. auris* clades. Phylogenetic analyses coupled with high-throughput phenotypic screening have shown that clades I and III are most closely related and display enhanced tolerance to osmotic stress, including high sodium chloride concentrations, compared to clades II and IV [109, 122]. Clade II isolates uniquely exhibit robust growth in the presence of sorbic acid and select dipeptide combinations, whereas isolates from all clades tolerate elevated levels of sodium nitrate, sodium phosphate, and ammonium sulfate. Across clades, *C. auris* grows efficiently on tricarboxylic acid (TCA) cycle intermediates such as citrate, succinate, and malate, but displays relatively poor growth on pyruvate, lactate, and acetate [123]. Chemical sensitivity profiling further identified six compounds—BAPTA, methyl viologen dichloride, protamine sulfate, thallium acetate, thiourea, and trifluoperazine—that inhibit growth across all clades [123]. Notably, trifluoperazine, an antipsychotic drug, and protamine sulfate, a heparin antagonist, are already approved for clinical use, highlighting opportunities for antifungal drug repurposing.

Clade-dependent differences also extend to antifungal drug susceptibility and responses to surface disinfectants [124]. Raman spectroscopy-based analyses revealed that amphotericin B induces biofilm formation in clades II and III, whereas exposure to 5-fluorocytosine promotes filamentation without biofilm development [125]. Although both clades increase ergosterol and chitin production in response to antifungal stress, clade III isolates uniquely reduce membrane permeability to 5-fluorocytosine by increasing chitin acetylation and elongating fatty acid chains. This adaptive response likely confers enhanced resistance to 5-flucytosine in clade III compared to clade II strains[125]. Structural differences in biofilm architecture further distinguish these clades, with clade II forming dense, continuous biofilms and clade III producing looser, discontinuous biofilms.

At the genetic level, clade-specific regulatory variation contributes to antifungal resistance. The transcription factor Mrr1 regulates azole resistance through control of the Mdr1 efflux pump [126–129] and also governs genes involved in methylglyoxal detoxification [130]. Elevated methylglyoxal levels, which occur in several disease states, have been associated with increased susceptibility to *C. auris* infection [130, 131]. Recent work demonstrated that Mrr1a, one of three *C. auris* Mrr1 homologs, mediates resistance to methylglyoxal and induces canonical Mrr1-regulated genes, including *MDR1* and *MGD1*, which encodes a methylglyoxal reductase [127, 132]. Notably, a clade III–specific *MRR1* allele encodes a hyperactive variant associated with increased methylglyoxal tolerance and enhanced azole resistance relative to other clades [132]. The transcription factor Upc2 further reinforces this resistance network by activating ergosterol biosynthesis genes and *MDR1* expression under Mrr1-dependent regulation [133].

Collectively, these studies underscore that clade-specific genetic, metabolic, and drug response differences profoundly shape the pathogenic potential of *C. auris*. Appreciating this diversity is essential for accurate outbreak reconstruction, antifungal susceptibility interpretation, and the development of clade-informed diagnostic, therapeutic, and infection control strategies.

## Emerging diagnostic approaches for *C. auris*

Delayed initiation of antifungal therapy is a major contributor to mortality in invasive candidiasis, underscoring the critical need for rapid and accurate diagnosis. Effective clinical management depends on timely identification of the causative pathogen and its susceptibility profile [134]. This need has driven the development of innovative diagnostic platforms, particularly point-of-care (POC) assays designed to accelerate detection, guide early therapeutic decisions, and support infection control efforts.

Several promising POC tools are under development for *C. auris*. These include assays that detect and quantify *C. auris*-derived β-1,6-glucan [135] and β-1,3-glucan [136] directly from blood samples. A portable droplet magnetofluidics-based POC platform has demonstrated high specificity in identifying isolates from major *C. auris* clades (South Asia, South America, and Africa) with no cross-reactivity to other clinically relevant *Candida* species, including *C. albicans*, *C. duobushaemulonii*, *C. glabrata, C. haemulonii, C. krusei,* *C. parapsilosis*, and *C. tropicalis* [137]. Another rapid diagnostic strategy integrates recombinase-aided amplification with lateral flow strip (RAA-LFS) detection, enabling discrimination of *C. auris* from closely related species, including *C. haemulonii*, *C. pseudohaemulonii*, and *C. duobushaemulonii*, within 15 min from diverse clinical specimens, including blood, urine, and nasal swabs [138]. In parallel, simplified single-tube POC PCR assays are being developed to provide rapid and accurate *C. auris* detection directly from patient samples [139].

Beyond POC platforms, specialized laboratory technologies are further enhancing *C. auris* identification and susceptibility profiling. MALDI-TOF MS has been widely adopted for reliable species-level identification of *Candida* isolates [140, 141], provided that reference databases are appropriately curated. More recently, MALDI-TOF MS has been adapted for AFST, enabling rapid assessment of *C. auris* susceptibility to echinocandins such as anidulafungin [8]. The ASTA MicroIDSys MALDI-TOF MS platform has similarly demonstrated high accuracy in identifying a broad spectrum of pathogenic yeasts in clinical microbiology laboratories [142, 143].

The expansion of MALDI-TOF MS from species identification to antifungal susceptibility assessment represents a significant advance, offering the potential to markedly shorten diagnostic turnaround times while supporting real-time epidemiological surveillance. When combined with established molecular or phenotypic detection models, MALDI-TOF MS-based approaches can enable high-performance, non-invasive classification of resistance profiles in *C. auris* isolates, thereby informing clinical decision-making and antimicrobial stewardship. Importantly, reliable differentiation of *C. auris* from closely related species remains essential for outbreak containment, allowing timely isolation of colonized patients and safe discharge of non-carriers in healthcare settings [144].

Together, these emerging diagnostic platforms hold considerable promise for transforming the clinical management of *C. auris* by enabling earlier detection, guiding targeted antifungal therapy, and strengthening outbreak surveillance and infection control strategies.

## Emerging therapeutic strategies for *C. auris* infections

The current antifungal arsenal is extremely limited, and *C. auris* presents a major therapeutic challenge due to its frequent multidrug resistance. Standard antifungal classes include azoles, which inhibit ergosterol biosynthesis via lanosterol 14α-demethylase (Erg11); polyenes, such as amphotericin B, which bind ergosterol and disrupt membrane integrity; and echinocandins, such as caspofungin, which inhibit β-(1,3)-D-glucan synthase, an essential enzyme for fungal cell wall synthesis. However, azole resistance is widespread [145, 146], amphotericin B is associated with significant host toxicity [147], and *C. auris* can remodel its cell wall to reduce echinocandin efficacy [148, 149]. These limitations have intensified efforts to identify novel antifungals, repurpose existing drugs, develop synergistic combinations, and explore host-directed and vaccine-based strategies (Table 1).

**Table 1:** Alternative compounds to standard antifungal drugs with reported activities against *C. auris*

Category	Compound/therapy	References
Growth inhibition	Ethanolic extract from Caryocar brasiliense	[197]
Growth inhibition	Ethanolic leaf extracts from Coccinia indica	[198]
Growth inhibition	Cis-diaminocyclohexyl group (compound 18)	[199]
Growth inhibition	Manogepix (active moiety of the prodrug fosmanogepix)	[200]
Growth inhibition	Disulfiram	[201]
Growth inhibition	Macrocyclic amidinourea BM1	[202]
Growth inhibition	Combination of valine isocyanide with N-formylvaline	[203]
Growth inhibition	Keto-alkyl-pyridinium molecules	[204]
Growth inhibition	Synthetic triazoles compounds (13, 20 and 27) containing benzyloxy phenyl isoxazole side chain	[205]
Growth inhibition	Synthetic triazoles molecules (16, 18, and 29) with alkynyl-methoxyl side chains	[206]
Growth inhibition	Synthetic miconazole-based azoles dihydroeugenol-imidazole 14 derived from eugenol	[207]
Growth inhibition	N-demethyltyroscherin and tyroscherin from deep-sea sediment strain Scedosporium apiospermum FKJ-0499	[208]
Growth inhibition	Natural compound NPD5296	[209]
Growth inhibition	Synthetic 1,3-diethylthioselenoglycoluril	[210]
Growth inhibition	Ibrexafungerp (SCY-078)	[211]
Growth inhibition	Bisphenylthiazoles (compounds 16 and 17)	[212]
Antibiofilm activity	Octenidine dihydrochloride (Ocd)-chitosan (Cs) bandage	[213]
Antibiofilm activity	Postbiotic elements derived from Lactobacillus paracasei 28.4 cells	[214]
Antibiofilm activity	Kinase inhibitor Bay 11–7085	[215]
Antibiofilm activity	Synthetic phenylthiazole small molecule (Compound 1)	[216]
Antibiofilm activity	Photodynamic therapy using visible lights (blue, red, green) with or without photosensitizers	[187]
Synergistic activity	Artemether and fluconazole	[217]
Synergistic activity	Anidulafungin coupled with manogepix or 5-flucytosine	[218]
Synergistic activity	Host defense peptide mimetic brilacidin	[219]
Synergistic activity	Taurine-induced silver ions (Tau-Ag) combined with itraconazole	[220]
Synergistic activity	Combined serotonin reuptake inhibitor sertraline and voriconazole	[221]
Synergistic activity	Posaconazole combined with atazanavir or saquinavir	[159]
Synergistic activity	Miconazole combined with domiphen bromide	[222]
Synergistic activity	HIV protease inhibitors (e.g., atazanavir, saquinavir, lopinavir, ritonavir) combined with amphotericin B	[223]
Vaccine candidate	Spontaneous nanoliposome antigen particles designed by employing fructose bisphosphate aldolase and methionine synthase	[224]
Vaccine candidate	VXV-01 (Als3p + Hyr1p with CAF01 adjuvant)	[225]
Nanomaterial-based growth inhibition	Synthetic superparamagnetic Fe_2_O_3_ nanoparticles	[226]
Nanomaterial-based growth inhibition	Anacardic acid-dimethylglyoxime-loaded zein nanoparticles (ZA3)	[227]
Nanomaterial-based growth inhibition	Silver nanoparticles based on curcumin cyclodextrins loaded into bacterial cellulose-based hydrogels (cAgNP-loaded BC)	[228]
Nanomaterial-based growth inhibition	Synthetic silver nanoparticles (AgNPs)	[229]
Nanomaterial-based growth inhibition	Silver functionalized nanostructured titanium	[230]
Nanomaterial-based growth inhibition	Moringa oleifera-stabilized silver nanocomposites (Ag–MO and Ag–Zn–MO)	[231]
Other	Antimicrobial peptide bsa001	[232]

### Repurposed and combination therapies

Drug repurposing has yielded several promising candidates with activity against multidrug-resistant *C. auris*. Nitroxoline, a well-tolerated oral antimicrobial, demonstrated potent antifungal activity across clinical isolates (MICs 0.125–1 mg/L) [150]. Other FDA-approved or clinically used agents – including pyrvinium pamoate (impaired mitochondrial structure, lowered tricarboxylic acid cycle enzyme function, and blocked *C. auris* replication within macrophages [151]), dihalogenated 8-hydroxyquinolines (dihalogenated at the C5 and C7 positions [150, 152, 153]), and mefloquine-derived aminoquinolines [153]—exhibit broad activity across clades, including efficacy in animal models of disseminated infection.

Combination therapy represents a complementary strategy to overcome resistance. Synergistic interactions have been reported for pyrimidine analogues-polyenes combinations and pyrimidine analogues-echinocandins, particularly flucytosine with amphotericin B or micafungin[154], as well as isavuconazole (≥ 0.125 mg/L) combined with ≥ 1 mg/L echinocandins [155]. Polyene-echinocandin combinations improve survival in invertebrate infection models [156]. Additional synergistic interactions include sulfamethoxazole restoring azole activity [157], oxindole efflux inhibitor (azoffluxine) enhancing efflux pump inhibition, decreasing fungal burden by ~ 1000-fold as a single agent, and improving *C. auris* susceptibility to azoles (fluconazole, [158]), and enhancement of antifungal efficacy by HIV protease inhibitors [159]. Notably, tacrolimus—a calcineurin inhibitor—exhibited strong synergy with itraconazole across all tested *C. auris* isolates, without antagonism [160]. Collectively, these findings support the rationale that targeting multiple cellular pathways can enhance antifungal efficacy and potentially delay resistance emergence, though clinical validation remains limited.

### Next-generation synthetic antifungals and clinical pipeline

Beyond repurposing, synthetic antifungal discovery has expanded substantially. Diverse chemical scaffolds—including β-nitrostyrenes, triazolylacetamides, and glutamine analogs –disrupt cell wall integrity, inhibit biofilm formation, or induce apoptosis in *C. auris* [161]. Notably, the glutamine analog Nva-FMDP selectively targets fluconazole-resistant isolates with elevated chitin content by inhibiting glucosamine-6-phosphate synthase [162]. However, rapid resistance evolution, including the emergence of mutator phenotypes linked to DNA repair defects, highlights the vulnerability of single-target monotherapies [162, 163].

Among translationally advanced candidates, fosmanogepix (FMGX) has demonstrated potent in vitro activity and promising clinical outcomes in phase II trials for *C. auris* candidemia, achieving high microbiological clearance and survival with favorable safety profiles [164]. These results underscore the feasibility of targeting novel fungal pathways, while emphasizing the importance of resistance-mitigating strategies.

### Natural products, nanotechnology, and physical approaches

Natural products remain a critical source of antifungal innovation. Compounds such as turbinmicin—targeting vesicular trafficking via Sec14—exhibit robust in vivo efficacy in murine models [165]. Other natural molecules, including eugenol, thymol, dehydrocurvularin, and plant-derived extracts, show antifungal and antibiofilm activity, though cytotoxicity and synthesis challenges limit some candidates [166–172]. Importantly, many frontline antifungals are themselves derived from natural products [173, 174].

Nanotechnology-based formulations aim to enhance drug stability, biofilm penetration, and therapeutic index. Amphotericin B nanoemulsions, alginate-encapsulated miltefosine, and phytofabricated silver nanoparticles demonstrate potent antibiofilm activity [175, 176]. However, clinical translation is constrained by concerns over bioaccumulation, immunogenicity, manufacturing complexity, and systemic delivery barriers [177–184].

Antimicrobial photodynamic therapy (aPDT) offers an alternative physical approach, using light-activated photosensitizers to generate reactive oxygen species and disrupt *C. auris* biofilms [185–187]. While effective for superficial infections, limited tissue penetration remains a key challenge for systemic application [188].

### Host-directed therapies and vaccines

Host-directed strategies provide an attractive adjunct to antifungal drugs. Human antimicrobial peptides—including β-defensin-3, histatin 5, and HNP-1—exert direct fungicidal activity while modulating innate immune responses [189, 190]. Immunomodulators such as Annexin A1 further limit fungal dissemination [191], and activation of pattern-recognition receptors enhances antifungal immunity.

Vaccine development represents a critical long-term strategy. Reverse vaccinology and epitope-based approaches have identified conserved antigens capable of eliciting cross-clade immune responses [192, 193]. The *C. auris*-specific adhesin Scf1, essential for colonization, biofilm formation, and virulence, emerges as a particularly promising vaccine or therapeutic target [194]. Although still preclinical, these efforts highlight the feasibility of durable immune protection against *C. auris*.

### Limitations and remaining challenges

Despite these advances, substantial barriers remain to the clinical translation of emerging therapeutic strategies for *C. auris*. Many promising agents and combinations are supported primarily by in vitro data or limited animal models, with few advancing to robust, controlled clinical trials. The extraordinary genetic plasticity of *C. auris*, including rapid acquisition of resistance mutations, aneuploidy, and mutator phenotypes, raises concerns that novel single-target agents may face rapid resistance emergence, particularly under monotherapy. Combination therapies, while conceptually attractive, introduce challenges related to toxicity, pharmacokinetic compatibility, drug-drug interactions, and patient tolerability. For natural products and nanotechnology-based approaches, issues of chemical complexity, scalability, formulation stability, long-term safety, and regulatory approval remain significant hurdles. Host-directed therapies and vaccines, though promising, must contend with variability in host immune status, risks of immunopathology, and the need for durable efficacy across diverse patient populations and fungal clades. Collectively, these challenges highlight the necessity of cautious optimism and emphasize that sustained progress against *C. auris* will depend on rigorous translational validation, resistance-aware drug development, and integration of therapeutic advances with improved diagnostics, infection control, and antifungal stewardship.

## Conclusions

*Candida auris* poses a formidable global health challenge due to its multidrug resistance, high mortality rates (30–60%), and persistence in healthcare environments. Its unique traits –including thermotolerance, halotolerance, and robust biofilm formation—facilitate colonization of human skin and hospital surfaces, hindering eradication efforts. Frequent misidentification with *C. haemulonii* and related species underscores the urgent need for improved diagnostic tools for rapid and accurate detection. Resistance to azoles, echinocandins, and polyenes—driven by biofilm formation, upregulated efflux pumps, and mutations in *ERG11*, *FKS1*, and *TAC1B*—further complicates therapeutic management.

Encouragingly, recent advances are expanding the therapeutic landscape. Repurposed drugs, rational combination therapies, and natural product–derived compounds are broadening antifungal options, while nanotechnology-based delivery systems, host-directed interventions, and multi-epitope vaccine strategies signal a paradigm shift in antifungal development. In parallel, genetic and functional tools—including CRISPR-Cas9-based approaches—are accelerating mechanistic insight into *C. auris* virulence, morphogenesis, and resistance, revealing new vulnerabilities that may be therapeutically exploited.

The rapid emergence and global dissemination of *C. auris* have catalyzed the adoption of integrated, multifaceted strategies that unite chemical innovation, bioengineering, and immune modulation. Sustained progress will require coordinated global surveillance, rigorous infection prevention measures, and multidisciplinary collaboration to translate mechanistic discoveries into effective diagnostics, therapeutics, and preventive interventions. Collectively, these efforts will be essential to curb the spread of *C. auris* and to establish durable defenses against this persistent and evolving fungal pathogen.

## Data Availability

No datasets were generated or analysed during the current study.
