# Correction: The emerging pathogen *Candida auris*: host interactions and disease drivers

**DOI:** 10.1186/s12929-026-01234-1

**Published:** 2026-03-18

**Authors:** Daniel Ruben Akiola Sanya, Ashley Valle Arevalo, Djamila Onésime, Clarissa J. Nobile

**Affiliations:** 1 INRAE, AgroParisTechMicalis Institute, Universite Paris-Saclay, 78350 Jouy‑en‑Josas, France; 2 Department of Molecular and Cell Biology, University of California, Merced, Merced, USA; 3 Health Sciences Research Institute, University of California, Merced, Merced, USA

**Correction: Journal of Biomedical Science (2026) 33:19** 10.1186/s12929-026-01230-5

After publication of this article [1], it was brought to our attention that the conflict of interest statement for the publication appears to be missing from the published paper.

The statement is: Clarissa J. Nobile is a cofounder and acting CEO of BioSynesis, Inc., a company developing diagnostics and therapeutics for biofilm infections.

The original article has been updated.
